# Novel DNA Topoisomerase IIα Inhibitors from Combined Ligand- and Structure-Based Virtual Screening

**DOI:** 10.1371/journal.pone.0114904

**Published:** 2014-12-09

**Authors:** Malgorzata N. Drwal, Jessica Marinello, Stefano G. Manzo, Laurence P. G. Wakelin, Giovanni Capranico, Renate Griffith

**Affiliations:** 1 Department of Pharmacology, School of Medical Sciences, UNSW Australia, Sydney, NSW, Australia; 2 Department of Pharmacy and Biotechnology, University of Bologna, Bologna, Italy; Concordia University Wisconsin, United States of America

## Abstract

DNA topoisomerases are enzymes responsible for the relaxation of DNA torsional strain, as well as for the untangling of DNA duplexes after replication, and are important cancer drug targets. One class of topoisomerase inhibitors, “poisons”, binds to the transient enzyme-DNA complex which occurs during the mechanism of action, and inhibits the religation of DNA. This ultimately leads to the accumulation of DNA double strand breaks and cell death. Different types of topoisomerases occur in human cells and several poisons of topoisomerase I and II are widely used clinically. However, their use is compromised by a variety of side effects. Recent studies confirm that the inhibition of the α-isoform of topoisomerase II is responsible for the cytotoxic effect, whereas the inhibition of the β-isoform leads to development of adverse drug reactions. Thus, the discovery of agents selective for topoisomerase IIα is an important strategy for the development of topoisomerase II poisons with improved clinical profiles. Here, we present a computer-aided drug design study leading to the identification of structurally novel topoisomerase IIα poisons. The study combines ligand- and structure-based drug design methods including pharmacophore models, homology modelling, docking, and virtual screening of the National Cancer Institute compound database. From the 8 compounds identified from the computational work, 6 were tested for their capacity to poison topoisomerase II *in vitro*: 4 showed selective inhibitory activity for the α- over the β-isoform and 3 of these exhibited cytotoxic activity. Thus, our study confirms the applicability of computer-aided methods for the discovery of novel topoisomerase II poisons, and presents compounds which could be investigated further as selective topoisomerase IIα inhibitors.

## Introduction

DNA topoisomerases I and II are essential enzymes that manipulate the topological states of DNA *in vivo* by cleaving and religating DNA strands in a controlled manner [1]. Their activity is critical to fundamental cellular processes such as transcription, replication, recombination, and DNA repair [2]–[4]. Mammalian cells express two isoforms of topoisomerase II (Top2) [5], [6]: Top2α predominates in actively replicating cells, its levels being modulated during the cell cycle, while Top2β is expressed constitutively, and is essential during particular cellular processes such as neural development [7], [8]. The principal function of Top2α is untangling DNA duplexes after replication, an activity achieved by introduction of double-strand breaks into DNA segments and transport of another DNA helix through the cut segments. Due to their pivotal role in cell viability, Top2 enzymes are important targets for several antitumour drugs [9]. By stabilizing the enzyme-DNA complex, the drugs lead to an apoptotic cascade of DNA double-stranded breaks, a so-called poisoning mechanism of action which is lethal for the cell [10], [11]. Whilst Top2 poisons have wide-spread clinical use, their utility is hampered by a variety of severe adverse side effects including cardiac myopathies and secondary malignancies [9], which appear to be correlated with their ability to poison Top2β [12], [13]. Therefore, the exploration of differences in structure and function between the two Top2 isoforms is important, and the development of selective Top2α inhibitors may be a beneficial strategy in the search for new cancer drugs with improved clinical safety.

The combination of structure- and ligand-based computational methods has been successfully applied in many drug design projects [14]. Pharmacophores, representing the spatial arrangement of chemical features necessary for binding of a small molecule to its biological target, can be developed in different ways, depending on the available information. Previously, we have used a combination of ligand- and structure-based pharmacophores as well as docking to identify novel inhibitors of topoisomerase I as potential anticancer agents [15], [16]. To test whether a similar approach could be used to identify novel topoisomerase II inhibitors selective for the α-isozyme, a combination of ligand- and structure-based methods was used in virtual screening and selected hits were tested *in vitro*.

## Results

### Ligand-based pharmacophore for Top2

Given the successful application of ligand- and complex-based pharmacophores and their combined use with docking methods in virtual screening to identify novel topoisomerase I inhibitors [15], a similar approach was chosen to identify novel topoisomerase II inhibitors. A set of 155 etoposide analogues, for which poisoning of the topoisomerase II-DNA complex has been measured using purified enzyme, was identified from the literature [17]–[34]. To the best of our knowledge, this set of compounds represents the only large set of molecules where topoisomerase II activities have been measured in the same laboratory using the same assay. The entire data set was used as a training set for the generation of ligand-based pharmacophores as described in the Methods section. The best pharmacophore hypothesis created from the input compounds contained 2 hydrogen bond acceptor (HBA), one cyclic π-interaction (CYPI) feature, a custom feature representing π-π-stacking interactions as described previously [15], as well as 5 excluded volumes indicating where no ligand atoms should be placed. The pharmacophore model showed high correlation between observed and predicted activities (r^2^ = 0.83) and high statistical significance (99%) and was therefore applied in virtual screening. However, due to the small number of features, the initial screen of the National Cancer Institute (NCI) database with the ligand-based etoposide pharmacophore resulted in a large number of hits (100338 compounds) and a further filter was necessary.

### Complex-based pharmacophore for Top2

**Figure 1:**
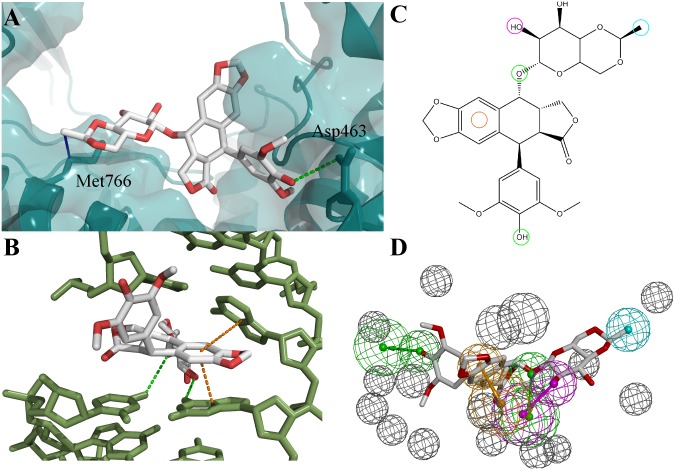
Docking pose of etoposide in Top2α and development of complex-based pharmacophore. A) Docking pose of etoposide relative to Top2α. Protein backbone and surface shown in cyan. Two residues interacting with etoposide are shown as sticks. B) Docking pose of etoposide relative to DNA. DNA residues shown as green sticks. Hydrogen bonds indicated as green, hydrophobic interactions as blue and stacking interactions as orange lines. C) and D) 2D- and 3D-representation of complex-based pharmacophore developed from the etoposide docking pose. Colour code of pharmacophore features: hydrogen bond acceptor (HBA), green; hydrogen bond donor (HBD), pink; hydrophobic group, blue; cyclic π-interaction (CYPI), orange; excluded volume, grey.

In a topoisomerase I molecular modelling study performed in our laboratory [15], ligand- and complex-based pharmacophores developed from the same chemical group of compounds were combined in virtual screening to reduce the number of hits. Therefore, a similar approach was used here. However, due to the lack of structural information on Top2α ternary complexes, a homology model of the human Top2α-complex was generated. Human Top2β is the closest homologue to Top2α for which the crystal structure of a DNA-ligand complex has been solved [35] and this was used as template for model development (see Methods). To generate a complex-based etoposide pharmacophore, the potential binding pose of etoposide in the Top2α-DNA complex was determined in docking experiments where docking settings had been optimized to reproduce the etoposide pose in the Top2β crystal structure. The best docking pose of etoposide in the Top2α-DNA complex displayed interactions with the residues Asp463, Met766 and with DNA residues at the cleavage site (Fig. 1). The observed interactions were translated into pharmacophore features and excluded volumes were added to represent the shape of the binding pocket. Application of the complex-based Top2 pharmacophore resulted in a reduction of the first hit list to 487 compounds, which was further reduced to 184 compounds by addition of a Lipinski filter (as described in the Methods section).

### Top2α versus Top2β pocket

**Figure 2:**
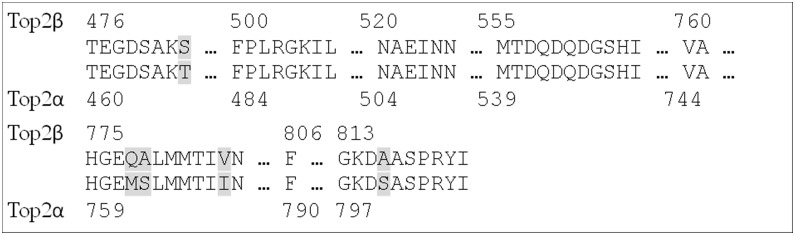
Sequence alignment of Top2α and Top2β ligand binding pocket residues. Non-conserved residues are highlighted.

**Figure 3:**
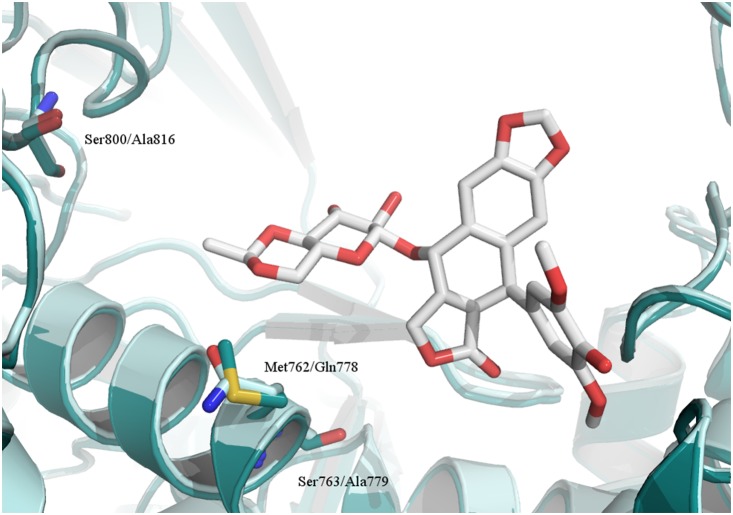
Possible residues to be targeted by Top2α versus Top2β-selective molecules. Superimposition of Top2α homology model (dark cyan) and Top2β crystal structure (light blue) [34], shown in cartoon representation. Residues in the etoposide binding pocket which differ between the Top2α and Top2β isoform are indicated as sticks (residue names: Top2α/Top2β). Etoposide pose from the Top2β crystal structure (stick representation, carbons white) shown for clarity.

The data used to develop the pharmacophores does not contain any information about discrimination between Top2α and Top2β. Therefore, the pharmacophores can be regarded as filters to focus the hit list towards Top2 inhibitors, but not a method to find Top2α-selective compounds. To investigate whether selective Top2α binding could potentially be achieved at the DNA cleavage site, the sequences of the α- and β-isoform at the binding sites were compared (Fig. 2). The analysis revealed that the binding site residues of Top2α and Top2β share high homology, with a sequence identity of 91.4%. In fact, only five of the binding pocket residues are not conserved between the two isoforms: Thr468/Ser483, Met762/Gln778, Ser763/Ala779, Ile769/Val785 and Ser800/Ala816 (α/β isoform). Threonine and serine are similar amino acids, and so are isoleucine and valine. Therefore, these residues might not be important for the development of selective inhibitors. However, methionine and glutamine have very different chemical properties – one being a hydrophobic, the other a polar amino acid. Additionally, serine and alanine differ in that serine is a potential hydrogen bond donor. The docking pose of etoposide in the Top2α model shows that Ser763 and Met762 are located in close proximity to etoposide and thus could be targeted by new inhibitors, whereas Ser800 might be too far away from the DNA cleavage site to be targeted by a Top2α poison (Fig. 3). It was thus investigated whether docking of the focused library developed above and filtering of compounds for Top2α-specific interactions can lead to the identification of Top2α-selective molecules.

### Docking of potential inhibitors

Compounds belonging to chemical groups already known to be Top2 inhibitors (acridines, anthraquinones and podophyllotoxins) were removed from the 184 compounds identified in pharmacophore screening. The remaining compounds were docked into the Top2α-DNA homology model using parameters optimized in control dockings. For all docking runs, a representative pose was extracted by finding the best-scoring pose of the largest cluster of solutions. The representative poses were analyzed according to their interactions with Top2α. Whereas none of the compounds was found to interact with Ser800, 12 molecules were found to form a hydrogen bond with Ser763 (Table 1). For comparative reasons, those 12 compounds were also docked into the Top2β binding pocket. Analysis of the docking poses revealed that two compounds, NSC 045569 and NSC 692660, showed a large number of interactions with the Top2β protein, in particular an interaction with Gln778, a residue only present in Top2β. Furthermore, compounds NSC 333772 and NSC 658583 exhibited higher docking scores in Top2β than in Top2α. Therefore, those 4 compounds were eliminated from the list of potential selective Top2α inhibitors and not investigated further.

**Table 1:** Compounds suggested from Top2α docking studies.

Compound	Docking score^1^	Mean score^2^	π-π-interactions (DNA)	Protein interactions
NSC 045569	88.61	78.38	6	7
NSC 333772	72.51	68.25	2	4
NSC 646829	74.54	69.10	6	4
NSC 647089	77.63	75.30	3	2
NSC 648591	81.60	69.60	3	5
NSC 648595	79.12	70.04	1	4
NSC 648628	75.40	68.29	5	2
NSC 658583	90.60	80.41	2	3
NSC 660823	88.51	79.17	2	2
NSC 660826	76.61	67.13	2	1
NSC 681699	70.58	67.06	1	3
NSC 692660	74.66	71.09	2	6

1 score of best-scoring pose of largest cluster;

2 mean score of largest cluster.

### *In* *vitro* Top2 assay

**Figure 4:**
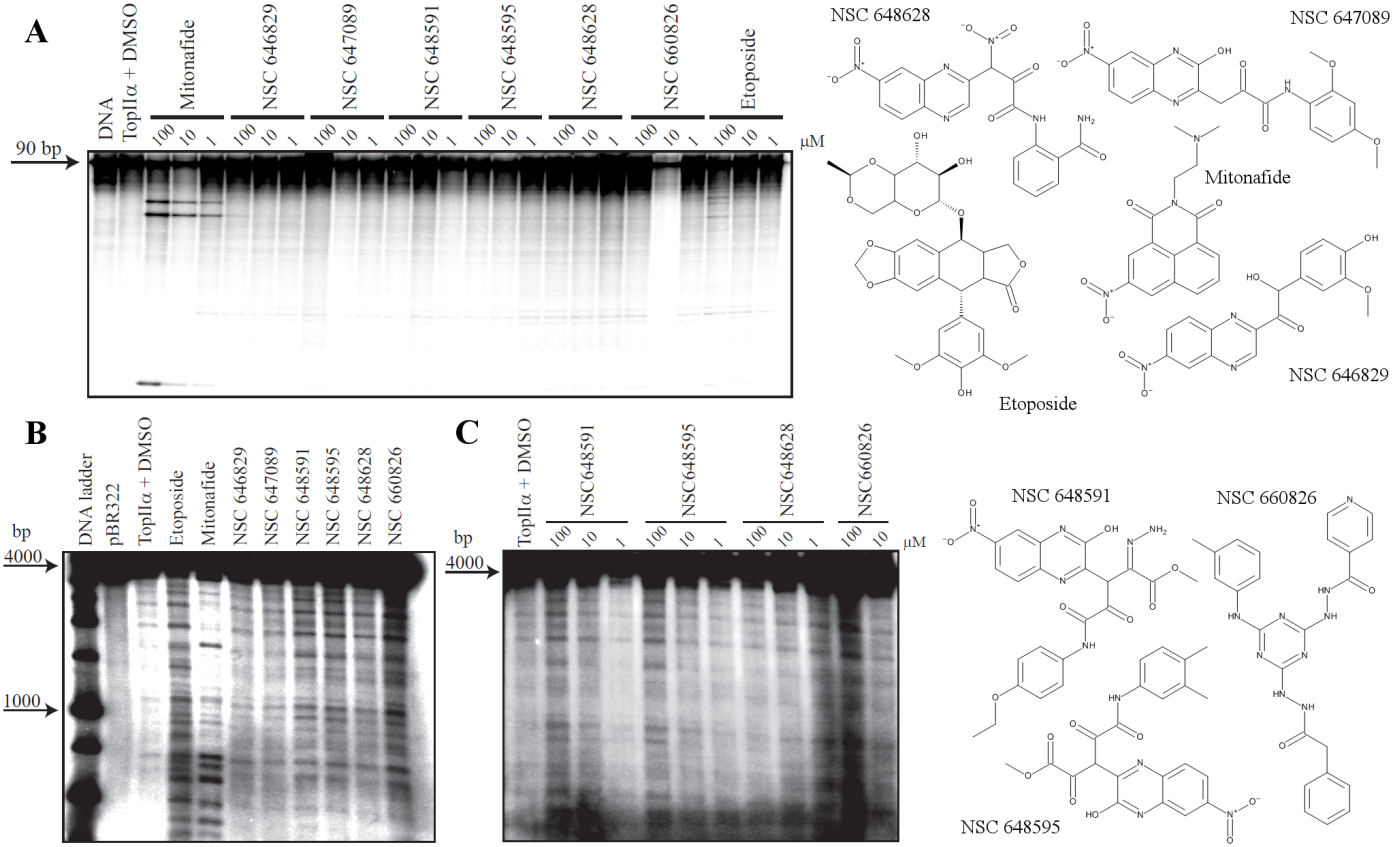
Topoisomerase 2α-mediated DNA cleavage by mitonafide, etoposide and *in silico* selected compounds. Structures of tested compounds and controls are shown on the right. A) Compounds were tested on a portion of SV40 sequence at three different concentrations (100, 10 and 1 µM). The first lane represents the DNA without enzyme and the second lane the DNA with enzyme in presence of DMSO (drug solvent). B) Same as in (A) but compounds were tested on pBR322 plasmid DNA at 100 µM concentration. C) The four active inhibitors of Top2α identified in (B) were additionally tested at 100, 10 and 1 µM concentration on pBR322 substrate.

To evaluate the ability of the 8 selected compounds to poison Top2α, specific *in vitro* DNA cleavage assays were performed. Due to the unavailability of NSC 660823 and NSC 681699, we were only able to test 6 of the suggested compounds (Table 2). Initially, the compounds were tested at high concentrations (100 µM) using the SV40 DNA portion as substrate and DNA fragmentation was analyzed by denaturing polyacrylamide gels. Mitonafide and etoposide were used as positive reference compounds for Top2α inhibition and they achieve cleavage stabilization in a dose dependent manner, in agreement with published data [36], [37]. In contrast to mitonafide, the selected compounds failed to demonstrate any ability to inhibit Top2α activity *in vitro*, as no prominent cleavage sites appear on the substrate after testing (Fig. 4A). Etoposide showed only weak inhibitory activity using the selected short SV40 DNA sequence [37]. As Top2 poisons can have high sequence selectivity, we then performed a DNA cleavage assay using the entire pBR322 plasmid as substrate and analyzed its fragmentation by agarose gel electrophoresis. Fig. 4B confirms the different sequence specificity of etoposide and mitonafide as the cleavage pattern is different between the drugs, even though both are efficient Top2α inhibitors. Four of the six tested compounds were found to be active as inhibitors of the enzyme at high concentration (100 µM), in particular NSC 648591, NSC 648595, NSC 660826 and to a minor extent also NSC 648628. In addition, all of them showed a cleavage pattern similar to etoposide, suggesting their mechanism of action could be similar to this drug, and further indicating that the employed SV40 DNA fragment is not a suitable substrate for testing potential inhibitors of unknown specificity.

**Table 2:** Compounds selected from screening with etoposide pharmacophores – biological results.

Compound (NSC)	CAS-RN^1^	Lowest GI_50_ (µM)^2^	Top2α inhibition
646829	907558-86-5	62.09	no activity
647089	907558-99-0	56.10	no activity
648591	Not found	24.21	activity at 100 µM
648595	Not found	18.54	activity at 100 µM
648628	908806-04-2	23.50	activity at 100 µM
660826	907548-30-5	inactive	activity at 100 µM

1 Chemical Abstracts Registration Number;

2 Cytotoxic activity measured in the US National Cancer Institute (NCI) 60 human tumour cell line anticancer drug screen [55], GI_50_ corresponds to the concentration of the drug resulting in a 50% growth inhibition.

**Figure 5:**
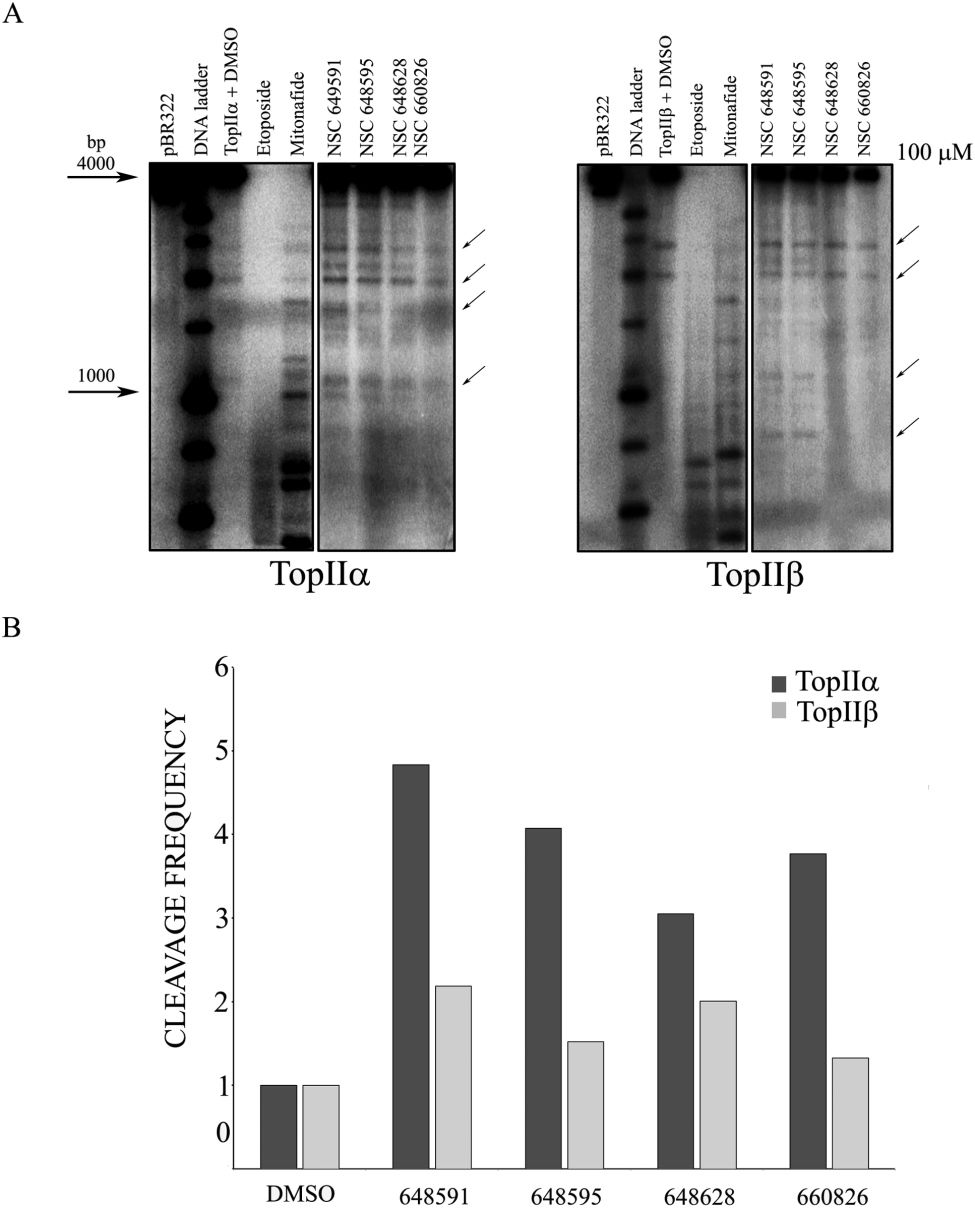
Top2α and β cleavage assays with selected compounds. A) The 4 active inhibitors of Top2α identified in Fig. 4B) were additionally tested at 100 µM concentration on both isoforms. The arrows show bands used for densitometric analysis. B) Preferential selectivity of selected compounds for the Top2α isoform. Semiquantitative analysis of cleavage product band densities was performed with Image Quant software (Molecular Dynamics). Data are reported from representative experiments as frequency of cleavage.

The inhibitory activity of the four compounds was additionally evaluated in a dose response cleavage assay using the pBR322 substrate, where the activity of NSC648591, NSC648595, NSC660826 and NSC648628 was minimal at the lowest concentration tested (10 µM; Fig. 4C). We then tested the activity of the active compounds against human Top2β to assess their isozyme specificity. Etoposide and mitonafide at 100 µM showed very high activity as the DNA was almost fully cut into smaller fragments that were not retained in the gel (Fig. 5A). At the same concentration, the four novel NSC compounds again inhibited Top2α weakly, while inhibiting Top2β to an even lower, if any, degree (Fig. 5A). The semiquantitative determination of drug-dependent increase of DNA cleavage by densitometric analysis confirmed that NSC648591, NSC648595, NSC660826 and NSC648628 were more effective against Top2α than Top2β (Fig. 5B).

These observations taken together suggest that four of the six selected compounds can be considered selective inhibitors of the Top2α enzyme.

## Discussion

Here, we present a combined ligand- and structure-based virtual screening study for the identification of novel topoisomerase IIα poisons. The computational methods include the integration of different types of pharmacophore models with comparative protein modelling and molecular docking. From the screening, 8 compounds were identified as novel potential selective Top2α inhibitors, 2 were not available for testing, and 4 were found to exhibit selective Top2α activity *in vitro*.

During the course of this project, a crystal structure of the human Top2α-DNA complex [38] has been solved. Although no ligand is present in this structure, the question arises as to whether the Top2α homology model developed here is comparable to the experimental structure of the *holo* enzyme. Therefore, we superimposed the predicted and experimental structures using a set of rigid residues identified with PROFLEX [39]. We found that the root-mean-square-deviation (RMSD) of all protein atoms is approximately 9Å. In particular, large differences between the model and the crystal structure are observed in the C-gate domain. These results are consistent with the findings of Wendorff and colleagues [38], who noted that the human Top2α structure shows differences to the Top2β-DNA-etoposide structure as well as the majority of topoisomerase II structures solved. Therefore, we conclude that the human Top2β crystal structure in complex with etoposide is a good template for homology modelling in the context of virtual screening and identification of novel inhibitors as it represents the protein conformation in the presence of a ligand. Further crystal structures of human Top2β-DNA-ligand complexes have also recently been solved [40]. The structures show the binding of the drugs amsacrine and mitoxantrone, two clinically used Top2 inhibitors, and exhibit high structural similarities to the Top2β-etoposide complex. These structures have not been taken into account in our study as only limited quantitative Top2α inhibition data was available for amsacrine and mitoxantrone analogues. However, they could be used in future studies to identify possible other Top2α inhibitors with binding modes different to etoposide.

Different DNA sequence preferences for the cleavage site bases have been reported for several topoisomerase II inhibitors [41], [42]. In this study, the four newly identified Top2α inhibitors showed a cleavage pattern similar to the known Top2α inhibitor etoposide, but not mitonafide. This is an interesting point as the entire computer-aided drug design methodology was based on etoposide analogues and the etoposide crystal structure. The Top2β-etoposide-DNA structure which was used as the template for homology modelling contains a DNA sequence cleaved between a cytosine and a thymine residue, the cytosine being the preferred DNA base for etoposide. The same DNA sequence was used in the docking experiments and thus had an influence on the selection of compounds for experimental testing. Therefore, it is perhaps not surprising that the compounds identified from virtual screening showed similar cleavage patterns to etoposide. Future studies could explore the effect of different DNA bases at the cleavage site of the modelled sequence on the docking results as well as the implications on the *in vitro* cleavage pattern.

The development of compounds selective for human topoisomerase IIα is desirable due to side effects arising from inhibition of the Top2β enzyme. To the best of our knowledge, no study has attempted to develop selective Top2α inhibitors which bind at the DNA cleavage site and the only selective inhibitors have been suggested to bind elsewhere [43]. This might be due to the high sequence similarity between the two isoforms, especially in the binding pockets. Nevertheless, the authors of the recently solved Top2α-DNA complex structure also suggest that Top2α-selective inhibitors could be designed by targeting the Top2α residues Met762 and Ser800 [38], confirming the validity of our approach. Although the four compounds identified show only modestly potent Top2α inhibition, three of the compounds exhibit clear cytotoxic activity (Table 2). In addition, all tested NSC compounds show selectivity for Top2α over Top2β and thus represent possible starting points for future development into novel selective Top2α inhibitors as anticancer agents.

## Methods

### Ligand-based pharmacophores

From the literature, a set of etoposide analogues was identified for which Top2 inhibitory activities have been measured in a K^+^/SDS precipitation assay for protein-DNA complex formation [17]–[34]. The assay used in all cases detects covalent protein-DNA complex formation in the presence of the drug, and the activity was expressed as a percentage in comparison to the amount of protein-DNA complex formation in the presence of etoposide (set to 100%). To be able to use the activity values in pharmacophore development, the percentages were transformed into an activity estimator using the average of known Top2 IC_50_ values of etoposide as a reference [42], [44], [45] and rescaled to span 4 orders of magnitude. Ligand-based pharmacophores were developed using the “3D QSAR Pharmacophore Generation” protocol in Discovery Studio 3.5 (DS; Accelrys, USA). Different parameters were evaluated, but the best correlation between observed and predicted activities was observed using an inter-feature distance of 3.5 Å, variable weights and tolerances, a maximum of 5 excluded volumes and the following pharmacophore features: hydrogen bond donors (HBD) and acceptors (HBA), cyclic π-interactions (CYPI) representing groups capable of stacking interactions [11], hydrophobic aliphatic (HYD) and positive ionisable (PI) features representing groups which are likely to be positively charged at physiological pH.

### Database screening

*In silico* database screening was performed using the DS protocol “Database Search” and the compound database of the National Cancer Institute (NCI2000) as implemented in DS. The number of hits was reduced by introduction of a Lipinski filter [46] allowing up to 1 exception. The DS protocol “Filter by Lipinski and Veber Rules” was used for this purpose.

### Homology modelling

To generate homology models of human Top2α, the crystal structure of the ternary Top2β-DNA-etoposide complex (PDB code: 3QX3 [40]) was used as a template. Multiple sequence alignments of Top2α, Top2β and related enzymes were performed using the ClustalW software [47]. The monomeric sequences of all enzymes were downloaded from the National Center for Biotechnology Information (NCBI) website (http://www.ncbi.nlm.nih.gov). The alignments were created using the BLOSUM scoring matrix and small manual adjustments were made by comparing the NCBI and PDB sequences.

Homology models were generated using the DS protocol “Build Homology Models” with dimer sequences. The protocol is an implementation of the Modeller program [48]. No symmetry constraints were used during the modelling of the two subunits. The top-scoring models were used. DNA molecules were copied from the templates and included in the homology models.

### Control docking of etoposide

Docking experiments were performed using GOLD [49], [50], accessed through the DS interface. Control dockings of the crystal structure ligands into their respective crystal structure were performed to test the docking settings. The binding sites were defined in DS based on the residues 10 Å around the crystal structure ligand position. Docking was performed either with a rigid protein or with flexible side chains. Side chains defined as flexible were selected based on their distance to the initial ligand position with a cut-off distance of 5 Å. This led to the selection of the following flexible residues: Glu477, Asp479, Ser480, Pro501, Leu502, Arg503, Gln778 and Met782 (Top2β). In the etoposide-Top2β-DNA complex, three water molecules were found to interact with etoposide and these three molecules were allowed to rotate, translate or disappear during the docking run.

After establishing the docking parameters in control docking runs, docking was performed into the best homology models of Top2α in complex with DNA. Flexible residues were defined according to the selection of flexible Top2β residues, which resulted in the following set: Glu461, Asp463, Ser464, Pro485, Leu486, Arg487, Met762, Met766 and Pro803.

### Complex-based pharmacophores

Complex-based pharmacophores were developed based on the best docking pose of etoposide in the Top2α homology model. The pharmacophore features were placed automatically using the DS protocol “Receptor-Ligand Pharmacophore”. The placed features were analysed carefully and checked for consistence with the interactions observed in the etoposide docking. CYPI features were added manually because the DS protocol does not allow the identification of custom features.

### Topoisomerase II-mediated DNA cleavage reactions

A duplex fragment of the SV40 genome which contains prominent topoisomerase II cleavage sites, positions 3449 to 3538, was purchased from Invitrogen Corporation (Carlsbad, CA) [51]–[53]. It was purified on a denaturing 20% polyacrylamide gel, each strand being recovered by ethanol-precipitation of water-soaked gel slices. Single-stranded DNA was 5′-labeled using T4 polynucleotide kinase (New England Biolabs, Ipswich, MA) with [γ-^32^P]ATP (3000 µCi/mmol, PerkinElmer Life and Analytical Sciences, MA) according to the suppliers’ instructions. Unincorporated nucleotides were removed using a QIAquick Nucleotide Removal Kit (Qiagen, Hilden, Germany). Radiolabelled duplex DNA was prepared by annealing with an equal amount of the non-radioactive complementary strand at 95°C, followed by slow cooling to room temperature. For the topoisomerase II cleavage reaction, DNA fragments were reacted for 30 min at 37°C in 40 mM Tris-HCl, pH 7.5, 80 mM KCl, 10 mM MgCl_2_, 5 mM DTT, 1 mM ATP, and 15 µg/ml bovine serum albumin with 30 units/sample of human topoisomerase IIα, purified as previously described [54], and the desired amount of drug. Reactions were stopped by adding 1% SDS and 0.2 mg/ml proteinase K and incubated at 42°C for 45 min. Samples were then ethanol precipitated, resuspended in 10 µl of formamide containing 0.25% bromophenol blue and xylene cyanol, heated at 95°C for 5 min, and chilled on ice. Reaction products were separated on 20% polyacrylamide denaturing sequencing gels. Dried gels were visualized using a B40 Storm phosphor imager (Amersham Biosciences, GE Healthcare, UK).

Plasmid DNA (pBR322, Carlo Erba Reagents, Milano, Italy) was digested with HindIII (Carlo Erba Reagents, Milano, Italy) and 5′-dephosphorylated with alkaline phosphatase (CIP, New England Biolabs, Ipswich, MA) before labeling with [γ-^32^P]ATP, as indicated above. For the topoisomerase II cleavage reaction, the same conditions were used for plasmid as for oligonucleotide DNA, but the plasmid DNA was electrophoresed in a 1.3% agarose gel in 89 mM Tris, 89 mM boric acid, 2 mM EDTA, pH 8, and 0.1% SDS.

Densitometric analysis was performed using Image Quant software (Molecular Dynamics). Briefly, the band intensities were determined by selecting fixed areas around bands. Local average background was calculated and removed from the quantification. Every cleavage product was normalized using uncut template density. The ratio between cut and uncut was calculated for 4 selected bands and the cleavage frequency was determined as the average of those ratios.
